# Task-Adaptive Angle Selection for Computed Tomography-Based Defect Detection

**DOI:** 10.3390/jimaging10090208

**Published:** 2024-08-23

**Authors:** Tianyuan Wang, Virginia Florian, Richard Schielein, Christian Kretzer, Stefan Kasperl, Felix Lucka, Tristan van Leeuwen

**Affiliations:** 1 Centrum Wiskunde & Informatica, Science Park 123, 1098 XG Amsterdam, The Netherlands; felix.lucka@cwi.nl; 2 Fraunhofer Development Center for X-ray Technology EZRT, Flugplatzstr. 75, 90768 Fürth, Germany; virginia.florian@iis.fraunhofer.de (V.F.); christian.kretzer@iis.fraunhofer.de (C.K.); stefan.kasperl@iis.fraunhofer.de (S.K.); 3 Mathematics Institute, Utrecht University, Campus-Boulevard 30, 3584 CD Utrecht, The Netherlands

**Keywords:** computed tomography, adaptive angle selection, defect detection, deep learning, reinforcement learning

## Abstract

Sparse-angle X-ray Computed Tomography (CT) plays a vital role in industrial quality control but leads to an inherent trade-off between scan time and reconstruction quality. Adaptive angle selection strategies try to improve upon this based on the idea that the geometry of the object under investigation leads to an uneven distribution of the information content over the projection angles. Deep Reinforcement Learning (DRL) has emerged as an effective approach for adaptive angle selection in X-ray CT. While previous studies focused on optimizing generic image quality measures using a fixed number of angles, our work extends them by considering a specific downstream task, namely image-based defect detection, and introducing flexibility in the number of angles used. By leveraging prior knowledge about typical defect characteristics, our task-adaptive angle selection method, adaptable in terms of angle count, enables easy detection of defects in the reconstructed images.

## 1. Introduction

X-ray Computed Tomography (CT) plays a pivotal role in non-destructive quality control, yet it remains predominantly an offline tool due to the trade-off between scan speed and scan quality. Standard CT protocols use evenly spaced angles around the object, typically requiring a large number of angles to ensure quality. However, in particular in sparse angle CT, the geometry of the object under analysis heavily influences which angular projections carry more or less useful information for the specific task to be performed, e.g., for defect detection [1,2]. Optimal Experimental Design (OED) aims to identify the optimal scanning angles to achieve a given task. We discern two primary categories: static OED and sequential OED. Static OED determines all experimental parameters in advance, without incorporating feedback from subsequent observations. In contrast, sequential OED relies on continuous feedback from each stage of the experiment, allowing for adjustments and adaptations based on emerging data. In the context of enhancing the speed of in-line CT inspections, the development of sequential OED for adaptive angle selection strategies is essential. Such a strategy would allow for a more focused scanning process, dynamically adjusting the angles and reducing the number of necessary angles, tailored to meet the specific needs of the inspection task. This approach promises to streamline the scanning process, making it quicker and more responsive to the unique characteristics of each inspection scenario.

Several researchers have employed prior information in the form of generic statistical models to determine the most informative angles using approaches based on OED [3,4,5,6]. However, computational challenges have limited the application of such methods to offline settings. In industrial applications, prior information in the form of computer-aided design (CAD) models is often available. Therefore, many researchers utilized them for offline training to determine the informative angles. The resulting predefined trajectories can then be rapidly applied in real settings. Fischer et al. [2] proposed a CAD-based method to optimize task-specific trajectories using a detectability index [7]. The index is computed by evaluating trajectory fitness against a user-defined frequency template, utilizing a modulation transfer function and the noise power spectrum. There are some extended works based on this: Herl et al. [8] considered a Tuy-based metric to guarantee the completeness of the data acquisition. Schneider et al. [9] used the regressive ResNet-34 to predict this index for each angle. Then, they used an integer optimization method to generate the trajectory based on these predictions. Matz et al. [10] proposed geometry-based metrics using a discrete wavelet transform to find which angle can capture more information about edges. Bussy et al. [11] employed the Q-Discrete Empirical Interpolation Method to obtain a subset from full angles. Furthermore, they introduced additional constraints considering the ray’s attenuation [12].

The need for adaptability in in-line use, particularly regarding angle selection, is underscored by the fact that these offline-optimized methods, while efficient in practical applications, often require precise alignment with actual scans [13]. To address this, we explored the potential of a Deep Reinforcement Learning (DRL) approach in the development of the sequential OED for adaptive angle selection in [14]. So far, we only considered approaches that choose a fixed number of angles to optimize generic image reconstruction quality measures.

This paper extends our previous work [14] and addresses the challenges of identifying defects in products using in-line CT. The contributions of this study include the following:

1. Incorporating prior knowledge about potential defects into the DRL reward function to guide task-specific angle selection for defect detection;
2. Acknowledging that defects in industrial products are infrequent, and that defective and non-defective samples may require different numbers of angles, our method introduces adaptability in angle count. This flexibility allows for a more detailed inspection when defects are suspected, enhancing defect detection while simultaneously optimizing the use of scanning resources;
3. Incorporating task-specific angle selection, such as image contrast or segmentation quality in the training reward. This integration of DRL with defect detection strategies significantly enhances both the rapidity and precision of defect identification, demonstrating the feasibility of an automated and efficient process for task-specific angle selection in CT scans.

This paper is structured as follows: The following section introduces the basic concepts and notation we use for the inverse problem in CT imaging and Reinforcement Learning (RL) for sequential OED. Section 3 elaborates on DRL and defect detection methodologies and outlines the integration of defect information into the RL reward function. Section 4 presents three experiments validating the algorithm. Section 5 discusses the advantages, limitations, and prospects for future research. This paper concludes in the final section with a summary of our findings.

## 2. Background

### 2.1. Inverse Problem of CT

In sparse-angle CT, we aim to reconstruct $\bar{x}$ from a small number of angles θ$\;=\left\{\theta_{1},\ldots ,\theta_{M}\right\}$, which presents an underdetermined and ill-posed inverse problem, which we will denote as

(1) $$y\left(\theta \right)=A\left(\theta \right)\bar{x}+\epsilon \left(\theta \right),$$

Traditional approaches like Filtered Back-Projection (FBP) are inadequate here, as they rely on a complete range of angles. We will use the Simultaneous Iterative Reconstruction Technique (SIRT) [15], which obtains a reconstruction by solving the constrained weighted least-squares problem

(2) $$\hat{x}\left(\theta \right)=\arg\min_{x\in Ʌ}{∥A(\theta )x-y(\theta )∥}_{R}^{2},$$

using a pre-conditioned projected gradient-descent scheme

(3) $$x^{\left(k+1\right)}=\Pi_{Ʌ}\left(x^{\left(k\right)}+CA{\left(\theta \right)}^{T}R\left(A\left(\theta \right)x^{\left(k\right)}-y\left(\theta \right)\right)\right).$$

In the above, $\Pi_{Ʌ}$ denotes a projection onto the constraint set (e.g., bound constraints $a\leq x_{i}\leq b$), the diagonal matrix *R* contains the reciprocals of row sums of A(θ) and the diagonal matrix *C* contains the column sums of A(θ).

### 2.2. Reinforcement Learning for Sequential OED

OED is a methodological framework developed to select experimental designs that maximize the information gained about parameters of interest [16]. In the example of (1), OED would aim to choose a set $\theta =\left\{\theta_{1},\ldots ,\theta_{M}\right\}$ such that y(θ) contains maximal information about a feature of $\bar{x}$. In sequential OED, as an extension, the experimental designs can be adapted sequentially based on the outcomes of previous experiments, e.g., we can choose each new angle $\theta_{k}$ based on the measurements $y(\left\{\theta_{1},\ldots ,\theta_{k-1}\right\})$.

While conventional OED formulations lead to complex optimization problems over utility functions measuring the information gain, we are considering using Reinforcement Learning (RL) to solve the sequential OED problem. RL is a machine learning paradigm in which intelligent agents learn to take actions in a dynamical environment that will maximize a reward they obtain [17]. RL is typically grounded in Markov Decision Processes (MDPs), a framework comprising action and state spaces, transition, and reward functions. RL algorithms aim to learn a policy—a strategy for selecting actions based on the current state—that maximizes cumulative rewards over time. Partially observable MDPs (POMDPs) extend this framework to scenarios, where the current state may not always be fully observable, and the agent has to construct a belief state that its actions are then based on.

We utilize RL within the POMDP framework to address the sequential OED problem following [18]. The RL algorithm operates by mapping the belief state at step *t*, denoted as $s_{t}$, to a probability distribution over the possible experimental designs or actions. The action for the next experiment, $a_{t}$, is then chosen based on this probability distribution. The objective is to learn a policy $\pi (a_{t}|s_{t})$ that optimizes the utility function of the sequential OED, essentially guiding the experimental design process in a way that maximizes the overall gain from the experiments conducted. Consequently, this methodology allows for the optimization of policy parameters rather than the design parameters themselves.

In [14], we described a POMDP model of the sequential OED problem of adaptively selecting a fixed number of scan angles in X-ray CT in terms of states, actions, and rewards associated with state transitions. The DRL training in this setup was focused on developing an optimal policy aimed at maximizing total rewards, primarily enhancing the reconstruction quality by choosing the most suitable actions based on the current state. The components of this model include:

- *Observation space*: it consists of a set of measurements y generated according to Equation (1).
- *State space*: we consider the reconstruction $\hat{x}$ of the underlying ground truth $\bar{x}$ as a belief state, which can be obtained via SIRT (3).
- *Action space*: it consists of 360 integer angles $\theta =\left\{\theta_{0},\theta_{1},...,\theta_{359}\right\}$ from the range [0, 360).
- *Transition function and observation function*: the transition function is deterministic, as the underlying ground truth remains unchanged. On the other hand, the data model given by the forward model serve as the observation function, from which we only consider measurement samples.
- *Reward function*: the reward function primarily evaluated the reconstruction quality using the Peak Signal-to-Noise Ratio (PSNR) value.

## 3. Methods

### 3.1. Task-Adaptive Angle Selection for Defect Detection

**Figure 1:**
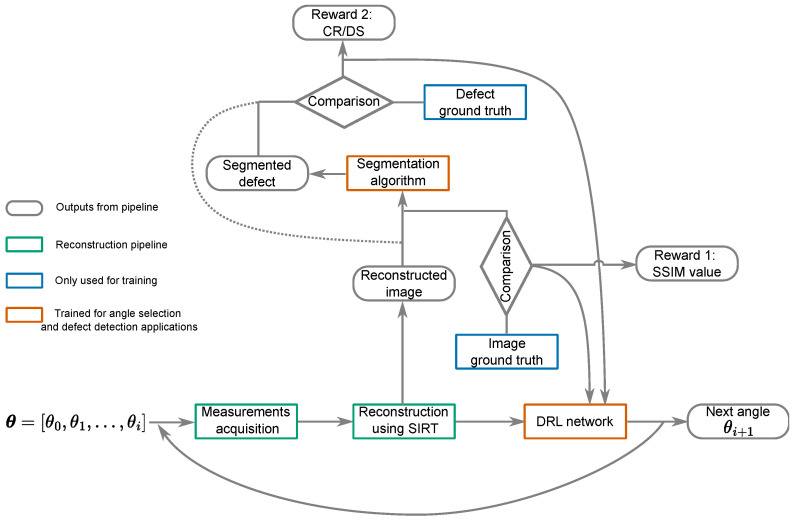
The DRL network selects the next angle $\theta_{i+1}$, which is used, along with the previously selected ones and measurements, to gather a new reconstruction. The SSIM is then calculated to assess the similarity between this reconstruction and the ground truth image. Concurrently, the contrast ratio value is derived from the ground truth of the defect (indicated by the dashed line); alternatively, the Dice score value is determined by comparing the defect segmentation output with the defect’s ground truth (depicted by the solid line). These metrics inform the DRL network’s decision-making for further angle selection.

Building on the approach outlined above, we now introduce a more complex reward function to facilitate adaptive, task-specific angle selection with a flexible number of angles. We propose an integrated pipeline where angle selection aids in improving defect detectability. As shown in Figure 1, the pipeline involves training two key components: the DRL network and the evaluation for defect detectability. The DRL network follows the architecture described in our previous work [14]. It includes a deep neural encoder network that extracts informative features from reconstructions at each step. Additionally, it incorporates an Actor–Critic network to estimate the value function and output the probability distribution over the action space. The bottleneck layer of the encoder network serves as the input to the Actor–Critic network.

The pipeline operates as follows: To explain one step of this sequential pipeline, we assume that a set of angles $\theta =\left\{\theta_{0},\theta_{1},...,\theta_{i}\right\}$ and their corresponding measurements are acquired. The SIRT algorithm with constraints is then used for image reconstruction. This reconstructed image serves as the input for the DRL network.

The reward function considers reconstruction quality, which is measured by comparing the reconstruction to the ground truth, often using the Structural Similarity (SSIM) index. It also evaluates defect detectability, described by metrics such as the contrast ratio (CR) or Dice score (DS) for segmentation precision. A negative reward is assigned at each step to encourage the use of fewer angles. More details about this reward function are provided in the following section.

The policy maps the input reconstructed image to a probability distribution over the action space, from which the next angle is selected by sampling. The policy is trained to maximize the total rewards and the computational costs spent on the training process.While the training process is computationally intensive, the trained model can be applied quickly in real-world applications.

### 3.2. Reward Function

The reward function considers two primary objectives: defect detectability and reconstruction quality. The detectability of defects, given the current reconstruction ${\hat{x}}_{k+1}$ and the ground truth of the defect $\bar{z}$, will be assessed by a measure $D\left({\hat{x}}_{k+1},\bar{z}\right)\in \left[0,1\right]$, where higher values mean a better detectability. We will describe two examples of $D({\hat{x}}_{k+1},\bar{z})$ in more detail in the following section. To asses reconstruction quality, we will use the SSIM index instead of the PSNR value, because the SSIM also takes values in the range [0, 1]. With this, the reward function is defined as

(4) $$R\left({\hat{x}}_{k+1},\bar{x},\bar{z}\right)=\left\{\begin{array}{cc} \alpha (SSIM\left({\hat{x}}_{k+1},\bar{x}\right)+D\left({\hat{x}}_{k+1},\bar{z}\right)) & \mathrm{if}\;\mathrm{stop}\;\mathrm{criteria}\;\mathrm{is}\;\mathrm{reached}\\ -a & \mathrm{otherwise} \end{array}\right.$$

The stop criteria are reached if the number of angles k+1 is equal to a maximum *M*, or if the reconstruction quality $SSIM({\hat{x}}_{k+1},\bar{x})$ is above a threshold *Q*. The rationale behind setting *M* is to prevent the process from selecting an excessively large number of angles, while the threshold *Q* ensures that angle selection stops in defect-free cases, where $D(\hat{x},\bar{z})=0$ at each step, and that the reconstruction quality is high enough for reliable defect determination. The term α is a scale. The term −a acts as a penalty at each step, encouraging the RL agent to select fewer angles to meet the stop criteria. Both the contrast ratio (CR) metric and Dice score (DS) metric can serve as the component $D({\hat{x}}_{k+1},\bar{z})$ of the reward function (4).

To effectively assess the detectability of defects, it is important to introduce specific metrics that quantify how clearly a defect is visible. The CR measures the visibility of a defect against the background material in terms of the average intensity difference, as follows:

(5) $$CR=\frac{|\mu \left(I_{\mathrm{defect}}\right)-\mu \left(I_{\mathrm{background}}\right)|}{\mu (I_{\mathrm{background}})}$$

In this formula, $\mu (I_{\mathrm{defect}})$ represents the mean intensity value of the defect area, while $\mu (I_{\mathrm{background}})$ denotes the mean intensity value of the surrounding background area. A greater CR value between a defect and the surrounding material often correlates with better detectability.

In practical terms, however, one must also take into account the actual segmentation of the defect. To this end, we explore unsupervised defect segmentation using the K-means clustering algorithm, a method celebrated for its uncomplicated yet effective approach to categorizing complex data into distinct clusters.

K-means clustering begins by determining a predetermined number of clusters, known as *K*, with each cluster represented by a central value or ’centroid’. The algorithm then proceeds in an iterative fashion, assigning each data point to the closest centroid using a distance metric, typically Euclidean distance. Centroids are recalculated in each iteration, and this process repeats until the centroid positions no longer change significantly.

Our paper leverages the K-means clustering algorithm to identify defects within material samples. By initializing the algorithm to create two clusters, we effectively distinguish between normal and defective regions. The defective cluster is then used to guide the manual segmentation of defects, illustrating the algorithm’s practical application in materials analysis.

When this semantic segmentation method is applied to the reconstructed image, one can also consider a metric that directly measures the accuracy of the segmented image. The DS compares the predicted segmentation of defects against the actual (ground truth) segmentation. It is expressed as

(6) $$DS=\frac{2\times |P_{\mathrm{predicted}}\cap P_{\mathrm{ground}\;\mathrm{truth}}|}{|P_{\mathrm{predicted}}\left|+\right|P_{\mathrm{ground}\;\mathrm{truth}}|}$$

Here, $P_{\mathrm{predicted}}$ refers to the set of pixels identified as defects in the predicted segmentation and $P_{\mathrm{ground}\;\mathrm{truth}}$ represents the set of pixels identified as defects in the actual ground truth segmentation. Thus, $P_{\mathrm{predicted}}\cap P_{\mathrm{ground}\;\mathrm{truth}}$ is the set of correctly identified defect pixels. The cardinality operator |·| measures the size of the sets, i.e., the number of active pixels.

These metrics offer insights into how effectively the chosen imaging angles can accentuate defects in the scans. By incorporating CR or DS into the reward function, we can systematically evaluate and optimize the angle selection process, ensuring that it maximizes the visibility and detectability of defects.

## 4. Results

In the results section of our paper, we explore the efficacy of the pipeline in selecting informative angles that enhance defect detectability. We compare our RL-based method to non-adaptive angle selection criteria. These non-adaptive angle selection criteria use the same number of angles, which is determined by the RL-based method. Our experimental analysis spans various datasets, ranging from synthetic numerical simulations to those that closely mimic real-world scenarios.

### 4.1. Baseline Angle Selection Methods

This subsection introduces the foundational angle selection methods used as baselines in our X-ray CT studies:

- Equidistant policy: This method is a conventional strategy for determining the acquisition angles in X-ray CT imaging. It uniformly divides the full 360-degree rotation into equally spaced angular intervals. With an increase in the number of angles, the interval between consecutive angles becomes narrower, offering denser angular coverage.
- Golden standard policy [19,20]: In contrast to the equidistant approach, this method adopts a non-uniform strategy for angle selection, leveraging the golden ratio for angle distribution. This ensures that with the addition of angles, the previously established angles remain fixed, and new angles are allocated within the largest existing interval between angles, according to the golden ratio. This dynamic adjustment can optimize angular coverage.

### 4.2. Shepp–Logan Phantoms

Initially, our experiment employs a numerical dataset and incorporates the CR metric into the reward function. The primary goal of this preliminary test is to demonstrate that our approach can effectively enhance defect detectability.

#### 4.2.1. Dataset

**Figure 2:**
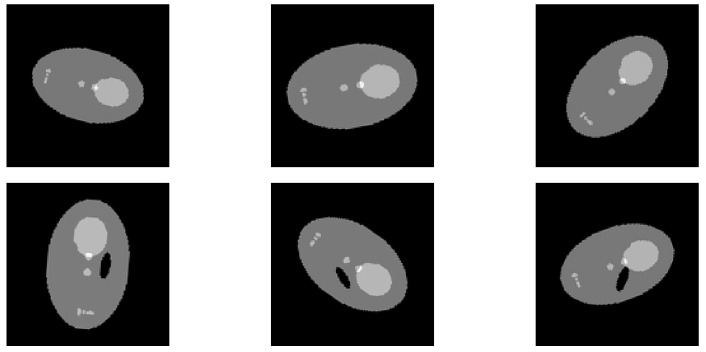
This figure displays samples of Shepp–Logan shapes, illustrating both normal (**top row**) and defective variants (**bottom row**). Each sample is unique, demonstrating a range of scales, shifts, and rotations, which reflects the diversity of the dataset used in our analysis.

For the first experiment, we generated images of objects consisting of ellipsoidal parts inspired by the Shepp–Logan phantom. The dataset consists of 1800 images of size 128×128, evenly divided into 900 normal samples and 900 samples with elliptical defects. The shapes and defects are characterized by random variations in scale and rotation. The Shepp–Logan shapes have scales within the following ranges: minor axis length between 57 and 67 pixels, and major axis length between 90 and 105 pixels. The defects have scales within these ranges: major axis length between 9 and 25 pixels, and minor axis length between 6 and 19 pixels. The rotations are distributed across 36 angles, each spaced equally from 0° to 179°. The diversity in the dataset is depicted in Figure 2.

#### 4.2.2. Implementation

When computing the CR value, we consider an elliptical background area slightly larger than the elliptical defect, with both its major and minor axes extended by four pixels. For the stop criteria, we set the maximum number of angles, M, to 20, the threshold for reconstruction quality, Q, to 0.85, scale α to 15, and penalty −a to −1. The Astra-toolbox [21,22] is employed for both forward projection and reconstruction tasks.

In the forward projection process, we utilize a fan-beam geometry. The distance from the source to the detector is set at 400 units, while the distance from the source to the object is 200 units. The detector is configured with a resolution of 256 pixels.

For the reconstruction phase, we employ SIRT with bound constraints, i.e., we run (3) with $Ʌ=\{x\in R^{n}|0\leq x_{i}\leq 1\}$ for 150 iterations.

#### 4.2.3. Evaluation

**Figure 3:**
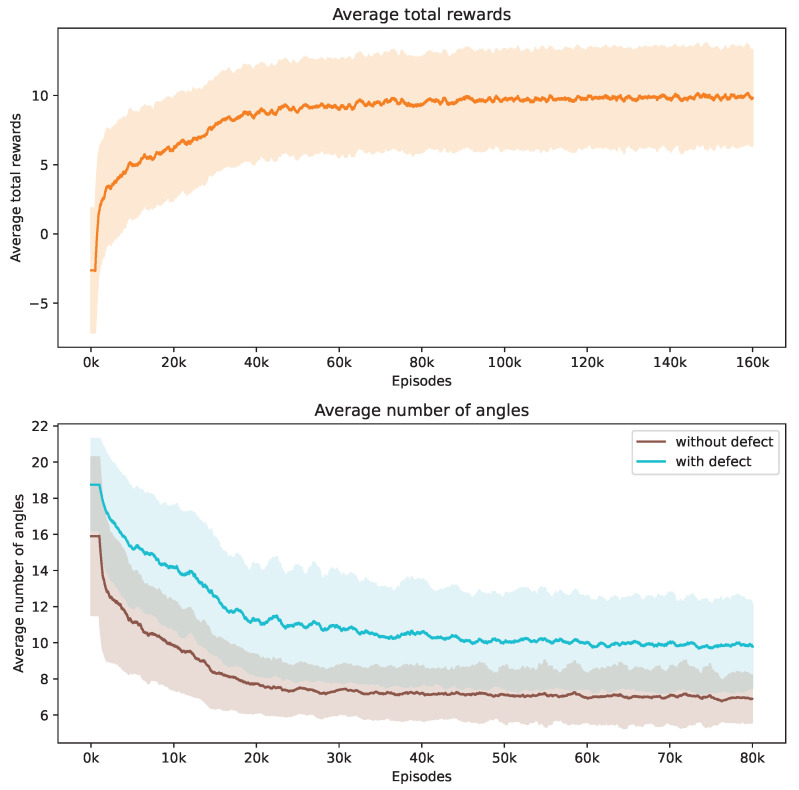
Evolution of average total rewards and the number of angles over training episodes. The figure at the top presents the trend in average total rewards achieved by the DRL agent throughout the training episodes. The figure at the bottom illustrates the variation in the average number of angles selected by the DRL agent for Shepp–Logan shapes, differentiated by the presence or absence of defects. Analyzed at intervals of every 1000 episodes, the graph shows mean values as a central curve, signifying the average reward at each point, with variance depicted as shaded bands around the curve, representing the standard deviation and the spread of reward values over time.

During our training process, we conduct a series of 160,000 episodes to train the DRL agent. Each episode involves using a Shepp–Logan shape from our dataset. In Figure 3, the top demonstrates the progression of average total rewards accumulated by the DRL agent during the training phase. This trend indicates that the policy effectively learned to balance improving SSIM and contrast ratio values while reducing the number of angles over time.

The bottom of Figure 3 showcases how the number of angles chosen by the DRL agent adjusts in response to Shepp–Logan shapes, both with and without defects. Initially, the DRL agent tends to select a higher number of angles to meet the stop criteria. It is observed that the shapes with defects consistently require more angles compared to the normal shapes from the start of training. As training progresses, the number of angles decreases for both scenarios due to the negative reward applied at each step. When considered alongside Figure 3, this trend suggests that the DRL agent is optimizing its angle selection strategy by choosing fewer yet more informative angles, thereby enhancing the total rewards earned. We incorporate the contrast ratio into the reward function, which influences the agent’s behavior when defects are present. In these cases, more angles are needed to enhance the visibility of flaws against the background. Notably, the data reveal that the defective Shepp–Logan shapes necessitate approximately 11 angles for effective analysis, compared to around 7 for non-defective ones.

**Figure 4:**
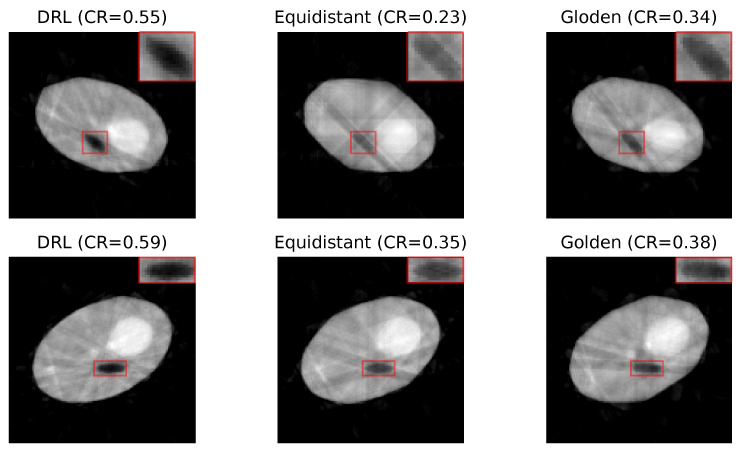
Reconstruction quality comparison. This figure presents a side-by-side comparison of reconstructed images obtained using the DRL policy versus those acquired through the equidistant and golden standard policies. The comparison highlights the differences in defect visibility across these methodologies. The numerical value accompanying each title denotes the CR value pertinent to its respective image, serving as a quantitative measure of the defect contrast compared to its surroundings. Red rectangles highlight the defect in each image, with a zoomed-in view displayed in the upper right corner to facilitate a more detailed examination of the defect visibility.

Figure 4 presents a set of reconstructed image samples obtained using the DRL, equidistant, and golden standard policies. The number of angles used in each row is determined by the DRL policy. The first row, utilizing 8 angles, and the second row, employing 10 angles, highlight the superior clarity of defects in images reconstructed by the DRL policy.

**Figure 5:**
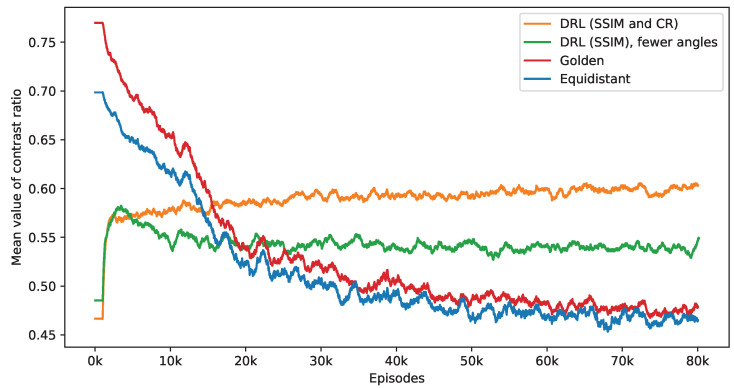
This figure presents a side-by-side comparison of CR values derived from different imaging policies. It specifically highlights the differences in CR values achieved by the DRL policy that utilizes both SSIM and CR as rewards, in contrast to the equidistant and golden standard policies. The number of angles selected by the DRL policy, which integrates SSIM and CR rewards, forms the basis for this comparison. The DRL policy informed by SSIM alone selects a smaller number of angles. It takes into account 1000 episodes, with the mean values representing the average. Additionally, calculations not depicted in this figure indicate that the DRL policy exhibits the smallest variance among these policies once the mean values reach a point of convergence.

The observed decrease in the number of angles chosen significantly influences the resulting CR values. Figure 5 contrasts the performance of three different policies—equidistant, golden standard, and DRL—in terms of their influence on CR value. The equidistant and golden standard policies utilize the number of angles determined by the DRL policy with SSIM and CR rewards. It is noted that while the average CR values under the equidistant and golden standard policies tend to diminish as the number of angles decreases, the CR values achieved using the DRL policy show a notable increase at the early stage and remain stable. Among these, the golden standard policy slightly outperforms the equidistant policy. Additionally, to evaluate the effectiveness of the CR reward, a separate DRL agent is trained solely with SSIM rewards. As shown in Figure 3, this approach initially leads to an increase in the CR values, which later decline. Upon reaching convergence, this SSIM-based DRL policy surpasses the equidistant and golden standard policies in performance but is still outperformed by the DRL policy, which incorporates a CR reward. Notably, the SSIM-based DRL policy focuses on a single task, leading to the selection of fewer angles than the DRL policy with dual tasks. Specifically, during the last 2000 episodes, the SSIM-based DRL policy selects an average of 8.07 angles, compared to 8.32 angles for the DRL policy with the CR reward. The DRL policy can handle variations in scale and rotation, ensuring reconstruction quality and effective defect detection with an appropriate number of angles. These findings suggest that including a reward for CR in the DRL framework aids in adaptively selecting angles that are not only optimal in quantity but also more effective for defect detection.

To assess the efficacy of the trained policy, we examine the same phantom subjected to previously unseen rotations. According to Table 1, the DRL policy with dual tasks demonstrates superior performance compared to two traditional approaches, across all policies considering the average number of angles, which is 8.46 as determined by the DRL policy. Additionally, both the golden ratio and equidistant policies require the selection of three additional angles to achieve performance comparable to that of the DRL policy with dual tasks, as indicated in the last two rows of Table 1.

### 4.3. Simulated Industrial Dataset

After the proof of the first experiment focusing on enhancing the contrast of the defect, a second experiment is carried out to assess the pipeline against a more realistic scenario and involve the defect segmentation.

**Figure 6:**
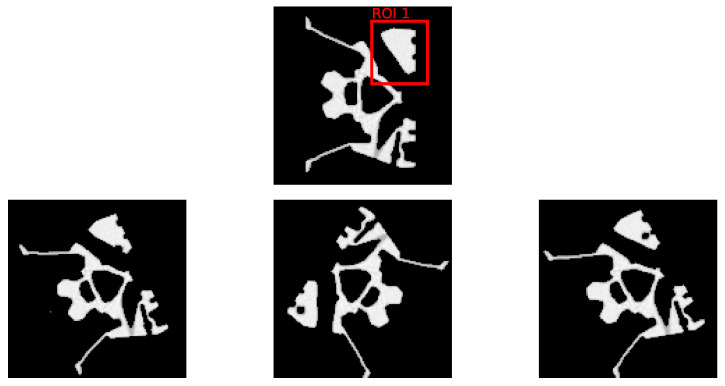
The **top row** depicts the selected ROI from the initial dataset, devoid of any defects. The **bottom row** presents three samples with artificially inserted pore defects within the ROI, each sample exhibiting a unique combination of rotation and scale variations to simulate defect diversity.

**Figure 7:**
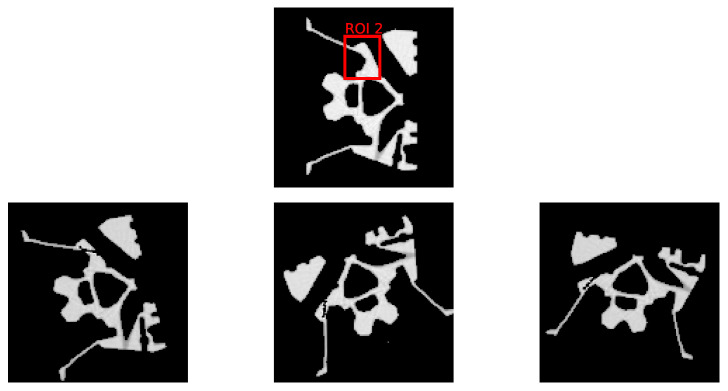
The **top row** depicts the selected ROI from the initial dataset, devoid of any defects. The **bottom row** presents three samples with artificially inserted crack defects within the ROI, each sample exhibiting a unique combination of rotation and scale variations to simulate defect diversity.

The Fraunhofer EZRT XSimulation software [23] is used to acquire a set of 1800 simulated images of size 128×128 in fan beam geometry. These simulated images serve as the ground truth and are used to compare with the K-means clustering segmentation to calculate the Dice score values. In this study, we focus on two prevalent defect types—pores and cracks—to evaluate the efficacy of the DRL approach. Within the domain of industrial CT scans, Regions of Interest (ROIs) are typically predetermined. Certain areas are more prone to defects and, consequently, demand a higher reconstruction quality. To reflect this industry practice, we designate two distinct ROIs to separately introduce pore and crack defects. Mirroring the setting of our initial experiment, we consider an equal distribution of normal and defective samples, totaling 900 each. Figure 6 and Figure 7 display examples of these artificially induced defects for reference. Variable position and tilt of the object are chosen for data augmentation and bias reduction.

In this research, we introduce pore defects of varying sizes into ROI 1. These defects encompass a pixel count between 11 and 35, with circumcircle diameters ranging from 3.2 to 7.0 pixels. This range presents diverse scenarios for the DRL method to tackle. Similarly, cracks are inserted into ROI 2, with a pixel count ranging from 7 to 25 and circumcircle diameters between 8.6 and 19.7 pixels. These experiments are designed to rigorously assess the DRL method’s effectiveness in segmenting defects and enhancing the quality of reconstructions within ROIs.

#### 4.3.1. Implementation

In this investigation, we assess the utility of our approach in augmenting the defect segmentation capabilities of the K-means clustering algorithm. Because the sample investigated in Section 4.3 has homogeneous materials, the number of clusters is two. Additionally, We identify the pixel with the maximum intensity value and use its position as the initial center for one of the clusters. This approach helps distinguish between the defect and non-defect areas. Finally, we compare the segmented results with ground truth annotations to validate the clustering performance using the DS metric.

To establish clear termination parameters for our process, we define two stopping criteria: a maximum count of 20 angles (M) and a minimum threshold for reconstruction quality within the ROIs, set at an SSIM of 0.45 (Q). To ensure uniformity throughout our experiments, we adhere to the same configuration settings used in our initial experiment, both in other parameters and within the Astra Toolbox. Additionally, we train two distinct DRL policies to address pore and crack defects, respectively, allowing for a targeted approach to each defect type and ROI.

#### 4.3.2. Evaluation

A. Pore defects

**Figure 8:**
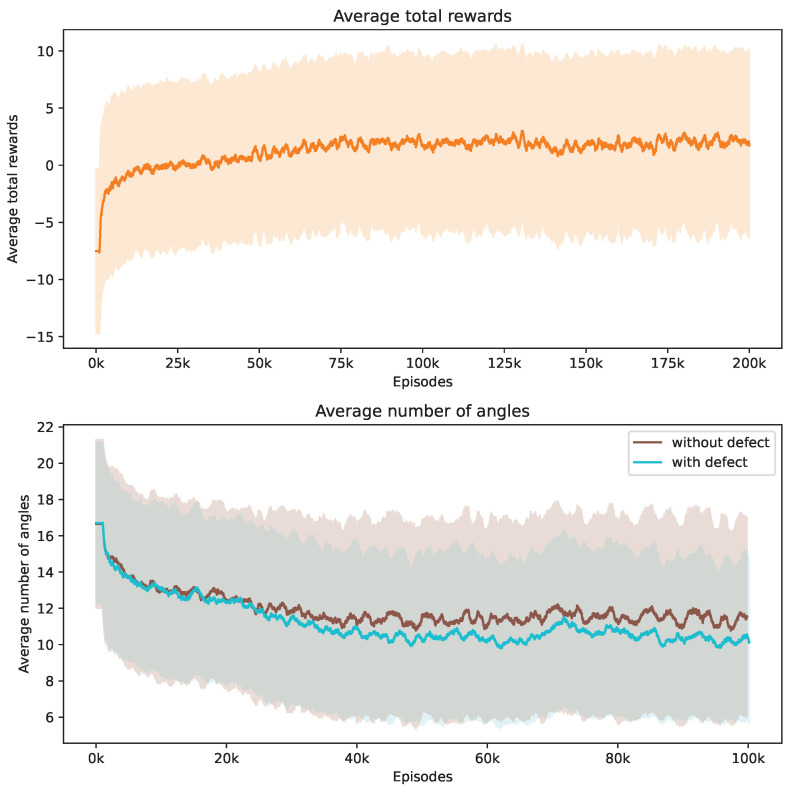
Evolution of average total rewards and the number of angles over training episodes. The figure at the top presents the trend in average total rewards achieved by the DRL agent throughout the training episodes. The figure at the bottom illustrates the variation in the average number of angles selected by the DRL agent for samples with ROI 1, differentiated by the presence or absence of defects. Analyzed at intervals of every 1000 episodes, the graph shows mean values as a central curve, signifying the average reward at each point, with variance depicted as shaded bands around the curve, representing the standard deviation and the spread of reward values over time.

Our investigation centers on pore defects within ROI 1. Figure 8 captures the evolution of the training phase, tracking both the average total rewards and the number of angles utilized. The upper section of Figure 8 demonstrates that the DRL agent successfully progressively increases its reward acquisition. The lower section reveals the strategy of the agent to minimize the number of angles used. Notably, non-defective samples tend to require a greater number of angles on average compared to defective ones, and they also exhibit a broader variance.

**Figure 9:**
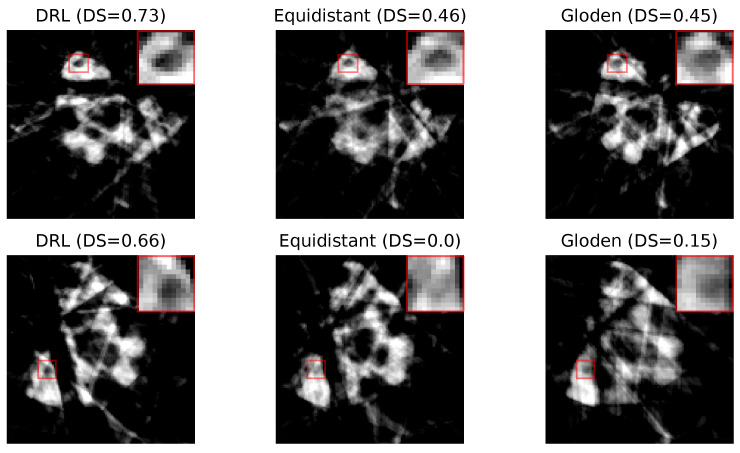
Reconstruction quality comparison. This figure presents a side-by-side comparison of reconstructed images obtained using the DRL policy versus those acquired through the equidistant and golden standard policies. The comparison highlights the differences in defect visibility across these methodologies. The numerical value accompanying each title denotes the DS value pertinent to its respective image, serving as a quantitative measure of the defect segmentation. Red rectangles highlight the defect in each image, with a zoomed-in view displayed in the upper right corner to facilitate a more detailed examination of the defect visibility.

Figure 9 presents two sets of reconstructed images using the DRL policy, alongside those derived from the equidistant and golden standard policies. This comparison is based on the number of angles determined by the DRL policy. The first row, utilizing 10 angles, and the second row, employing 7 angles, are evident that the reconstructions produced by the DRL policy are superior, demonstrating enhanced defect visualization. This improvement is corroborated by the corresponding DS values, indicating a higher accuracy of defect detection.

**Figure 10:**
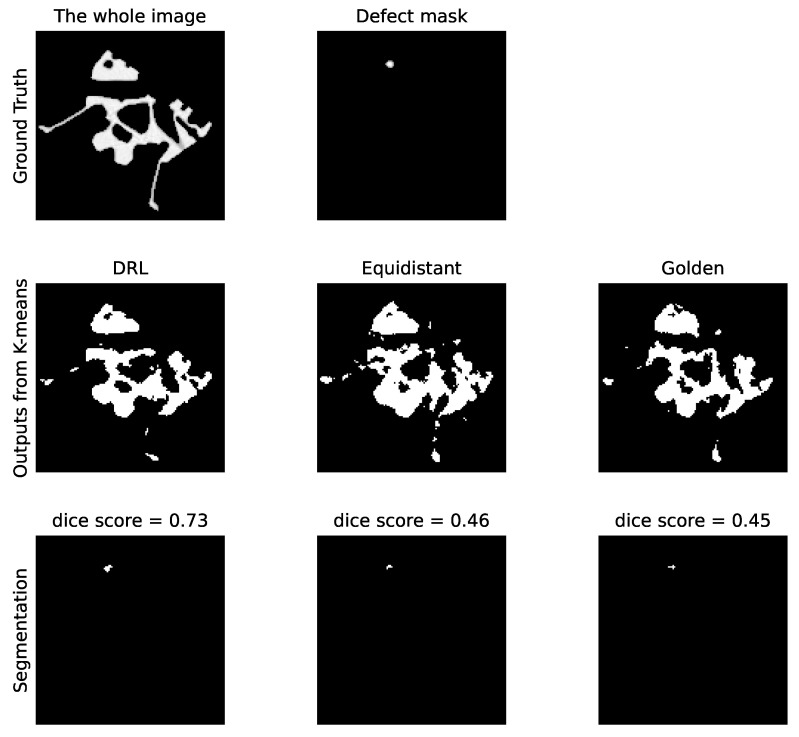
Comparative analysis of the pore defect segmentation. The top row represents the original image with ground truth defects. The middle row illustrates segmentation outputs using three different policies: DRL policy, equidistant policy, and a golden standard policy. The bottom row displays the corresponding defect masks generated by K-means clustering. The values of the DS for each method are indicated, quantifying the accuracy of the defect segmentation relative to the ground truth.

Subsequently, Figure 10 showcases the segmentation outcomes corresponding to the top group of reconstructions from Figure 9. The reconstructed images are processed via the K-means clustering algorithm, resulting in binary representations of the defects. Segmentation accuracy is then ascertained by comparing these binary images against the ground truth defect masks, demonstrating the practical effectiveness of our DRL policy in identifying pore defects within the examined ROI.

**Figure 11:**
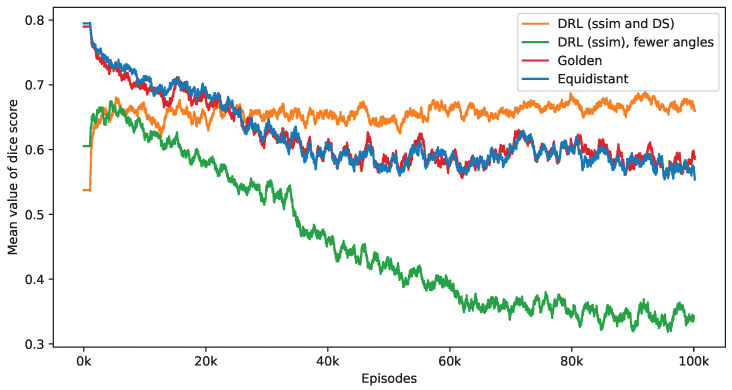
This figure presents a side-by-side comparison of DS values derived from different imaging policies. It specifically highlights the differences in DS values achieved by the DRL policy that utilizes both SSIM and DS as rewards, in contrast to the equidistant and golden standard policies. The number of angles selected by the DRL policy, which integrates SSIM and DS rewards, forms the basis for this comparison. The DRL policy informed by SSIM alone selects a smaller number of angles. It takes into account 1000 episodes, with the mean values representing the average. Additionally, calculations not depicted in this figure indicate that the DRL policy exhibits the smallest variance among these policies once the mean values reach a point of convergence.

Figure 11 presents the impact of reducing the number of selected angles on the DS value. This figure provides a performance comparison of three distinct policies—equidistant, golden standard, and DRL. The number of angles used by these policies is set according to the DRL policy, which integrates both SSIM and DS rewards. It is observed that while the DS values for the equidistant and golden standard policies generally decrease with a reduction in the number of angles, the DRL policy either sustains or enhances its DS values. Notably, the golden standard policy demonstrates a performance level that is similar to that of the equidistant policy. The effectiveness of the DRL policy, integrating both SSIM and DS rewards, is clearly demonstrated. In contrast, a DRL agent trained exclusively with the SSIM as its reward displays inconsistent performance, initially showing an increase in DS values but subsequently declining. When this training process reaches its endpoint, the SSIM-based DRL policy is outperformed by both equidistant and golden policies. As mentioned in the first experiment, the SSIM-based DRL policy necessitates fewer angles, averaging 5.65 over the final 2000 episodes, in contrast to the DRL policy that integrates both SSIM and DS rewards, which averages 10.84 angles. This difference in the number of angles is a contributing factor to the lesser performance observed in the SSIM-based policy. These findings validate the importance of including a DS reward in the DRL algorithm, significantly enhancing the selection of angles for improved defect segmentation. Without this reward, the DRL approach tends to focus solely on the reconstruction quality within the ROI while overlooking the specifics of the defect.

In a manner analogous to the experiments conducted on the Shepp–Logan phantom, we evaluate the trained DRL policy with dual tasks using rotations that are not included in the training set. The DRL policy selects an average of 9.84 angles. Table 2 shows that this policy outperforms two traditional policies in terms of performance. Furthermore, the last two rows of Table 2 indicate that both the golden ratio policy and the equidistant policy require the selection of two additional angles to achieve performance comparable to that of the DRL policy with dual tasks.

B. Crack defects

**Figure 12:**
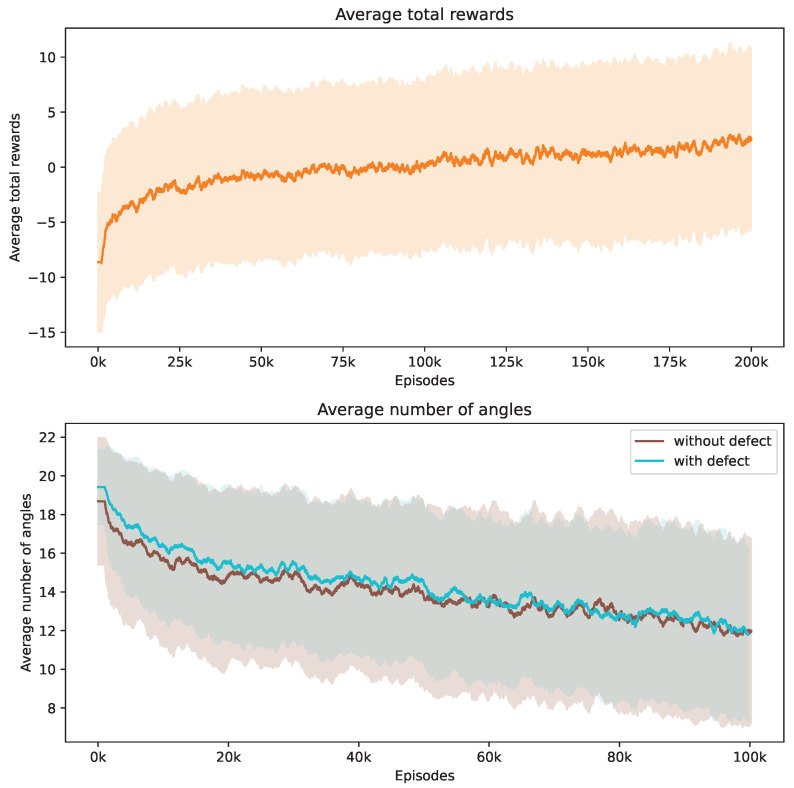
Evolution of average total rewards and the number of angles over training episodes. The figure at the top presents the trend in average total rewards achieved by the DRL agent throughout the training episodes. The figure at the bottom illustrates the variation in the average number of angles selected by the DRL agent for samples with ROI 2, differentiated by the presence or absence of defects. Analyzed at intervals of every 1000 episodes, the graph shows mean values as a central curve, signifying the average reward at each point, with variance depicted as shaded bands around the curve, representing the standard deviation and the spread of reward values over time.

Crack defects, with their distinctively long and thin shapes, pose unique challenges for automated detection and segmentation systems. In an experiment analogous to that conducted for pore defects, we observe that the average total rewards for the crack defect samples increase over time while the average number of angles used decreases, as shown in Figure 12. Interestingly, the number of angles required for accurately detecting defective samples is similar to that for non-defective samples in the case of crack defects.

**Figure 13:**
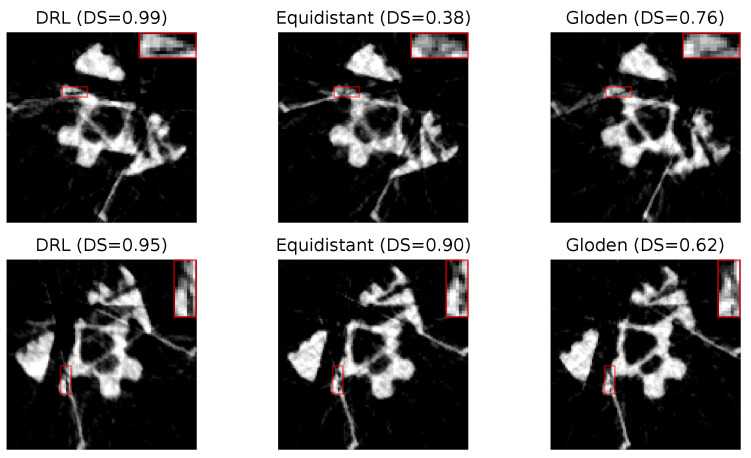
Reconstruction quality comparison. This figure presents a side-by-side comparison of reconstructed images obtained using the DRL policy versus those acquired through the equidistant and golden policies. The comparison highlights the differences in defect visibility across these methodologies. The numerical value accompanying each title denotes the DS value pertinent to its respective image, serving as a quantitative measure of the defect segmentation. Red rectangles highlight the defect in each image, with a zoomed-in view displayed in the upper right corner to facilitate a more detailed examination of the defect visibility.

In our analysis, Figure 13 displays the reconstructed images of crack defects using three distinct policies: the DRL policy, and the equidistant and golden standard policies. Additionally, the number of angles is determined by the DRL policy. The first row utilizes 14 angles, while the second row employs 18 angles. Similar to pore defects, the DRL policy, as indicated by the visibility of the defects and their corresponding DS values, appears to significantly enhance the segmentation process, making cracks more distinguishable for subsequent analysis.

**Figure 14:**
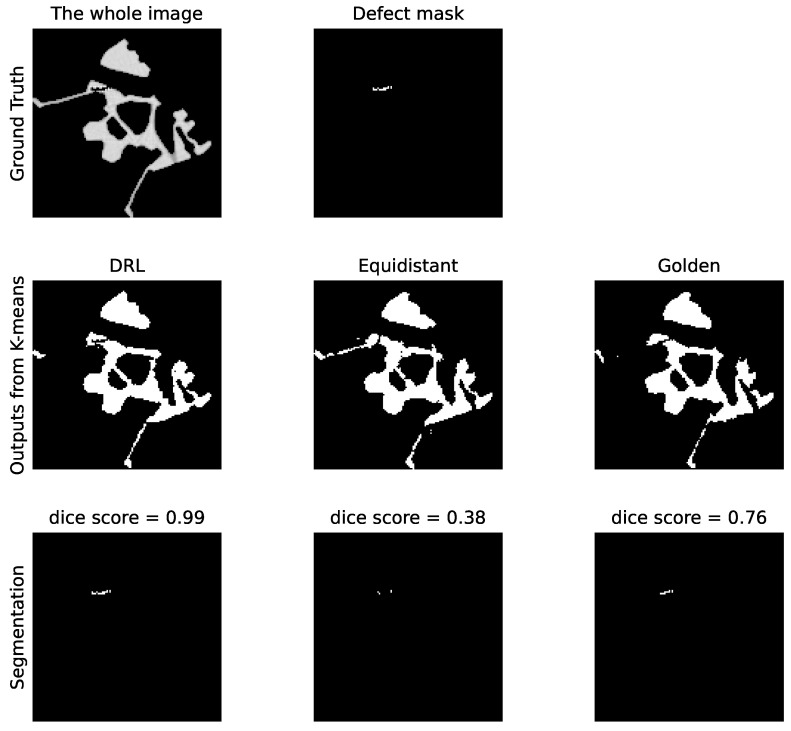
Comparative analysis of the crack defect segmentation. The **top row** represents the original image with ground truth defects. The **middle row** illustrates segmentation outputs using three different policies: DRL policy, equidistant policy, and a golden standard policy. The **bottom row** displays the corresponding defect masks generated by K-means clustering. The values of the Dice score for each method are indicated, quantifying the accuracy of the defect segmentation relative to the ground truth.

**Figure 15:**
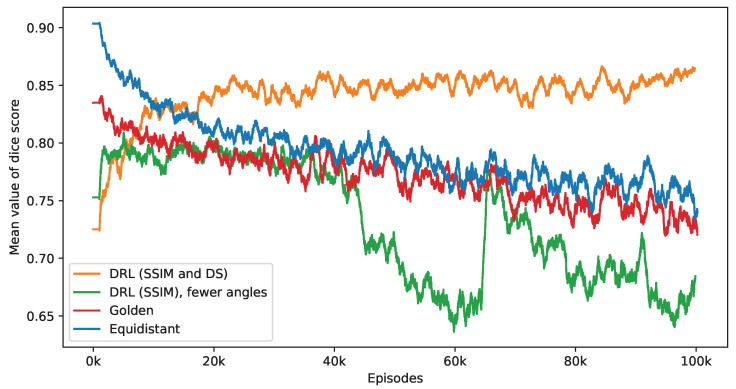
This figure presents a side-by-side comparison of DS values derived from different imaging policies. It specifically highlights the differences in DS values achieved by the DRL policy that utilizes both SSIM and DS as rewards, in contrast to the equidistant and golden standard policies. The number of angles selected by the DRL policy, which integrates SSIM and DS rewards, forms the basis for this comparison. The DRL policy informed by SSIM alone selects a smaller number of angles. It takes into account 1000 episodes, with the mean values representing the average. Additionally, calculations not depicted in this figure indicate that the DRL policy exhibits the smallest variance among these policies once the mean values reach a point of convergence.

Figure 14 presents the outcomes of applying the K-means clustering algorithm for segmenting these crack defects. The outcomes correspond to the first row in Figure 13. As evidenced by the DS values, the segmentation performance illustrates the DRL policy’s superior ability to accurately define and isolate the cracks within the material sample, underscoring its potential in improving segmentation in CT imaging. Additionally, Figure 15 presents a similar impact of reducing the number of selected angles on the DS value to Figure 11. When examining the last 2000 episodes, the average number of angles determined by the DRL policy, which incorporates both SSIM and DS rewards, is 11.86. In contrast, the average for the DRL policy that relies solely on SSIM is 9.39. However, it is noteworthy that the performance of the SSIM-based DRL policy exhibits a degree of instability.

In the context of a simulated industrial dataset featuring crack defects, the DRL policy with dual tasks selects an average of 12.26 angles. As indicated by Table 3, this policy surpasses the performance of two traditional policies. Additionally, the last two rows of Table 3 reveal that to match the performance of the DRL policy with dual tasks, both the golden ratio policy and the equidistant policy necessitate the selection of six additional angles.

## 5. Discussion

Our study began with numerical experiments using the Shepp–Logan phantom, featuring ellipse-shaped defects, and employed a CR-based reward to initially evaluate how defect information influences the DRL policy. Advancing towards more complex scenarios, we tackled the challenge of detecting smaller defects, which are inherently more difficult to identify. Our investigation encompasses two predominant defect types—pores and cracks—and incorporates K-means clustering for the segmentation of these defects. Following this, we integrate the DS metric into the reward function, showcasing its effectiveness in boosting the K-means clustering algorithm’s ability to segment defects. The findings reveal that DRL can successfully learn task-specific sequential OED policies that surpass traditional equidistant and golden standard approaches. This achievement underscores the potential to include additional tasks in our methodology, allowing for more tailored angle selection adaptations. In such cases, carefully optimizing the assigned weights for these tasks is crucial for achieving balanced and effective results.

Another notable aspect of our approach is the introduction of a negative reward at each step, encouraging the DRL agent to select an optimal number of angles. This strategy balances experimental cost and quality, proving advantageous for practical applications. The experimental results consistently surpass the outcomes of traditional equidistant and golden standard methods, underscoring the DRL approach’s ability to effectively select a flexible number of angles for improved defect detection.

To apply our method to real-world settings, the DRL approach has to be extended to incorporate one more component: to focus on task adaptation, a simple stopping criterion was used in this work, which depended on the ground truth images. In real-world settings, the DRL policy also needs to decide when to stop. One of the approaches for this that we will investigate in the future is to use the value function of the DRL framework, which is an estimate of future total rewards, as a stopping criterion, e.g., by setting a tolerance level for the output of the value function [24].

Several challenges and future works need to be addressed to advance this method. CAD models would be valuable for offline training of the DRL agent. However, it is essential to build a reliable simulation environment using CAD models that closely mimics real-world conditions. Apart from the metrics involved in the reward function, such as those assessing reconstruction quality and defect detectability, other metrics for industrial quality control can also be considered. Then, we aim to extend our method to encompass 3D geometries, in particular the cone-beam geometry, which is most commonly used in industrial applications. We also plan to investigate more dynamic scanning trajectories, including out-of-plane angles and variable focus, to align with the advanced capabilities of robotic CT scanners in industrial inspections. These developments promise to increase the versatility and applicability of our approach across diverse industrial inspection scenarios. Another aspect worth considering is the impact of the reconstruction algorithm on the efficiency of inline CT scans. Exploring deep-learning-based reconstruction methods [25] instead of traditional iterative methods like SIRT could potentially speed up the process, leading to more efficient industrial CT applications. Additionally, more efficient training methods, particularly reducing the computational complexity for handling large-scale data, need to be considered. Finally, adopting more advanced defect segmentation methods could improve our ability to manage the complexities associated with diverse defect types and imaging conditions.

## 6. Conclusions

This paper aims to expand the complexity of the reward function used in DRL for sequential OED and validate its effectiveness through various experiments. The inclusion of a reward for defect information enables the DRL agent to effectively learn task-specific angle selection. Moreover, our method offers flexibility in the number of angles utilized, achieving comparable reconstruction quality and defect detectability with fewer angles than those required by the golden ratio and equidistant policies. This methodology shows promise for online application in practice following the training phase.

In conclusion, our research sets the stage for future advancements in applying DRL to various industrial CT scanning scenarios, with the potential to improve the accuracy and efficiency of automated inspection technologies.

## Figures and Tables

**Table 1:** Comparative performance of DRL policy with dual tasks and traditional angle selection policies on Shepp–Logan phantom.

Policy	Average Number of Angles	CR	SSIM
DRL	8.46	0.62 ± 0.10	0.86 ± 0.01
Golden ratio	8.46	0.48 ± 0.13	0.83 ± 0.01
Equidistant	8.46	0.49 ± 0.17	0.83 ± 0.01
Golden ratio	8.46 + 3	0.61 ± 0.11	0.85 ± 0.01
Equidistant	8.46 + 3	0.59 ± 0.13	0.86 ± 0.01

**Table 2:** Comparative performance of DRL policy and traditional angle selection policies on the simulated industrial dataset with pore defects.

Policy	Average Number of Angles	CR	SSIM
DRL (9.84)	9.84	0.65 ± 0.24	0.46 ± 0.03
Golden ratio	9.84	0.57 ± 0.32	0.43 ± 0.09
Equidistant	9.84	0.56 ± 0.34	0.43 ± 0.07
Golden ratio	9.84+2	0.67 ± 0.25	0.47 ± 0.07
Equidistant	9.84+2	0.67 ± 0.28	0.47 ± 0.06

**Table 3:** Comparative performance of DRL policy with dual tasks and traditional angle selection policies on the simulated industrial dataset with crack defects.

Policy	Average Number of Angles	CR	SSIM
DRL	12.26	0.85 ± 0.14	0.46 ± 0.03
Golden ratio	12.26	0.72 ± 0.28	0.39 ± 0.10
Equidistant	12.26	0.76 ± 0.25	0.40 ± 0.10
Golden ratio	12.26+6	0.83 ± 0.18	0.49 ± 0.07
Equidistant	12.26+6	0.86 ± 0.14	0.49 ± 0.07

## Data Availability

Data are contained within the article.
